# Impact of COVID-19 Outbreak on Healthcare Workers in Italy: Results from a National E-Survey

**DOI:** 10.1007/s10900-020-00845-5

**Published:** 2020-05-22

**Authors:** Carla Felice, Gian Luca Di Tanna, Giacomo Zanus, Ugo Grossi

**Affiliations:** 1 Medicine 1 Unit, Treviso Regional Hospital, University of Padua, Treviso, Italy; 2 Statistics Division, The George Institute for Global Health, UNSW, Sydney, Australia; 3 IVth Surgery Unit, Treviso Regional Hospital, University of Padua, Piazzale dell’Ospedale, 1, 31100 Treviso, Italy; 4 Centre for Neuroscience, Trauma and Surgery, Queen Mary University of London, London, UK

**Keywords:** COVID-19, Survey, Coronavirus, SARS-CoV-2, Healthcare workers, Personal protective equipment

## Abstract

Italy has been the first-hit European country to face the outbreak of coronavirus disease 2019 (COVID-19). Aim of this survey was to assess in depth the impact of the outbreak on healthcare workers (HCW). A 40-item online survey was disseminated via social media inviting Italian HCW, with questions exploring demographics, health status and work environment of respondents. A total of 527 were invited to take part in March 2020, of whom 74% (n = 388) responded to the survey. Of these, 235 (61%) were women. HCW were mostly physicians (74%), from high-prevalence regions (52%). 25% experienced typical symptoms during the last 14 days prior to survey completion, with only 45% of them being tested for COVID-19. Among the tested population, 18 (18%) resulted positive for COVID-19, with 33% being asymptomatic. Only 22% of HCW considered personal protective equipment adequate for quality and quantity. Females and respondents working in high-risk sectors were more likely to rate psychological support as useful (OR, 1.78 [CI 95% 1.14–2.78] P = 0.012, and 2.02 [1.12–3.65] P = 0.020, respectively) and workload as increased (mean increase, 0.38 [0.06–0.69] P = 0.018; and 0.54 [0.16–0.92] P = 0.005, respectively). The insights from this survey may help authorities in countries where COVID-19 epidemic has not yet broken out. Management strategies should be promptly undertaken in order to enhance safety and optimise resource allocation.

## Introduction

The coronavirus disease 2019 (COVID-19) pandemic is affecting more than 200 countries and territories around the world, with a case fatality rate of 7% [1].

Since 21 February 2020, Italy has become the worst-hit country, with the highest death toll of 17,127 (over 5 times as much as China) on April 7th 2020. It has been shown that Italy has a higher proportion of older patients with confirmed COVID-19 infection than China and that the older population may partly explain differences in case-fatality rates between the two countries [2]. Other reasons may lay in different screening policies, hospital overcrowding, limited bed capacity in intensive care units compared to other European countries, relative delay from the first case detection on 21 February to the first containment decree from the government that closed the relevant villages, and stochastic factors determining high-prevalence foci in relatively small geographic areas [3].

In Italy, profound differences exist in terms of organization and management strategies, resulting in heterogeneous levels of performance across regional health systems. Since the early 1990s, a strong decentralization policy has been adopted, leading to the devolution of power to a lower governance level that is currently exercised in 21 regional health systems. Starting from 2015, all regions have been affected by the cutback initiative imposing to implement a gradual, phased reduction of the regional costs of personnel to be achieved before the end of 2020, even for the regional systems that manage to achieve a non-negative economic result [4]. Such a centrally-imposed manoeuvre has crystallized gaps in service provision and health system performance between regions. As a consequence, activation of different local protocols for the management of COVID-19 emergency (e.g. screening policy, indication to hospital admission, resource optimization) may have potentially contributed to the heterogeneity of observed outcomes even between neighbouring regions.

As a matter of fact, the prevalence of infection among healthcare workers (HCW) exceeds 10% in Italy [5] leading to further loss of capacity for hospitals to respond. In Lombardy, the worst-hit region, COVID-19 became largely a nosocomial infection [3]. To complicate matters further, experiences in Italy have demonstrated shortage of personal protective equipment (PPE) [6], which includes gloves, medical masks, goggles or a face shield, and gowns, as well as for specific procedures, respirators (i.e., N95 or FFP2 standard or equivalent) and aprons.

How Italian HCW are facing this challenging emergency contextualized in their own working environment is yet to be investigated by qualitative research. The aim of this study was to thoroughly explore the impact of COVID-19 outbreak on HCW in Italy and to provide useful hints for health authorities in order to tailor infection control strategies.

## Materials and Methods

A link for email registration was disseminated via social media (i.e. Twitter, Facebook, and LinkedIn) to capture HCW as potential respondents to the survey. The link was also advertised by the *Agenzia Nazionale Stampa Associata* (ANSA; literally “Associated Press National Agency”), the leading wire service in Italy [7].

Respondents were invited by an email to join a fully anonymous online closed survey (further details provided below). A total of 4 further email reminders (as per software restrictions) were sent throughout the period of online availability of the survey. Links expired after survey completion thus avoiding multiple responses by the same user. Ethical approval for this study was waived by the Ethics Committee for Clinical Experimentation (CESC) Marca Trevigiana, as institutional review board.

### Survey

A 40-item survey (namely, “Impact of COVID-19 outbreak on HCW in Italy”) was designed and developed by the first and senior authors (cf. & UG) using an online platform (“Online surveys” [formerly BOS-Bristol Online Survey], developed by the University of Bristol) in accordance with the Checklist for Reporting Results of Internet E-Surveys (the CHERRIES statement) [8]. Co-authors piloted the survey, assessed the design and checked the feasibility and validity of the questions. The finalized online survey was made available online from March 25th to April 4th 2020.

**Fig. 1:**
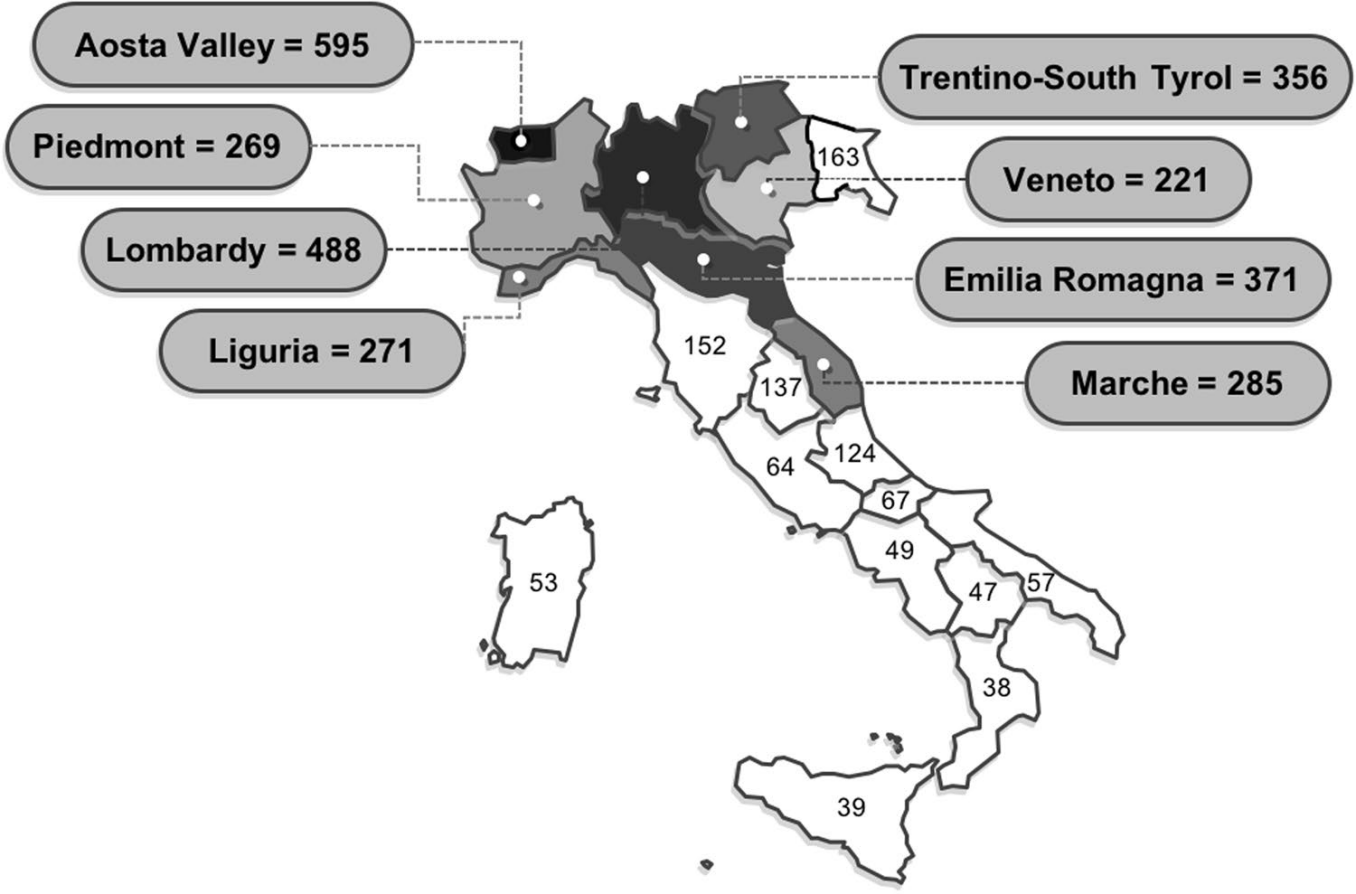
Regional prevalence of COVID-19 cases per 10^5^ people on survey closing date (April 4th 2020). Trentino-South Tyrol region includes both autonomous provinces of Trento and Bolzano

The survey aimed to assess crucial elements in HCW’s experience, and to capture key information about the respondents, including gender, age group, any health problems requiring chronic drug therapy, region of practice [using an arbitrary lower cut-off of 200 cases per 10^5^ people (according to the most updated national report) [9] to define regions with high prevalence on the survey closing date (Fig. 1)], type of HCW (e.g. physicians, nurses), employment contract, and workplace (e.g. academic hospital, non-academic hub or spoke hospital, general practice clinic, private clinic), and specialty sectors, which were further stratified into high- or standard risk for infection.

Six sectors were labelled as high-risk and included intensive care unit, pneumology, infectious diseases, emergency medicine, microbiology, and radiology. Other questions assessed the presence of COVID-19 positive subjects that respondents might have come in contact with; whether HCW were involved in the extraordinary management of COVID-19 patients with tasks beyond their own specialty; indication for COVID-19 screening at the workplace; whether HCW experienced typical symptoms (e.g. fever, dry cough, myalgia) in the past 14 days or were tested for COVID-19 exploring the prevalence of positive results. If testing positive, the use of personal protective equipment (PPE) and any medical therapy was explored. All respondents were asked whether they were quarantined or received influenza vaccine in 2019-20; whether they believed to have been the source of infection for patients, colleagues at work or family members. Quantity and quality of PPE at workplace was further explored as was how the number of intensive care unit beds changed from before the COVID-19 emergency till the outbreak; and if increased, whether implementation of dedicated staffing was also obtained. The activation of local protocols for management of COVID-19 patients was investigated and respondents asked whether they personally contributed to its development and/or complied with its requirements. Fatality related to COVID-19 was explored in terms of causality (i.e. exclusively resulting from respiratory failure or rather consequent to suboptimal bed capacity or non-compliance to protocols). Psychological impact of COVID-19 on HCW was also assessed in terms of psychological safety, experience of deaths among acquaintances, availability of psychological support at the workplace, and degree of workload. Finally, respondents were asked whether they could keep up with the medical literature on COVID-19.

All questions were set as mandatory fields with real-time validation and automated skip logic to prevent missing data and avoid illogical or incompatible responses. Quantitative data were automatically collected by the software and exported to a tabulated format.

### Statistical Analysis

Continuous variables were summarized by means and standard deviations, whilst categorical variables were assessed by proportions, with 95% confidence intervals (CI) calculated by Wilson score method with continuity corrections. Comparisons of categorical variables across groups were made by Pearson’s chi-square tests. A series of mixed models for continuous variables and hierarchical logistic models for binary variables were performed to assess the association between respondents’ preferences and their characteristics, with regions at high- and low prevalence of COVID-19 as random effects (adjusted odds ratio [OR]). Uni- and multivariable models were fitted using a pre-defined set of covariates which included age group, sex, regional prevalence, risk sectors, and type of HCW.

Non-hierarchical models were fitted to quantify the associations with regions at high- and low prevalence of COVID-19.

The denominator of the percentages of respondents was the total number of HCW who eventually completed the survey. Adjustment to the P-values was not performed. However, critical appraisal of P-values < 0.05 was conducted to take into account multiple testing and minimize the risk of false positives.

All analyses were performed using STATA 16 (StataCorp LLC, College Station, TX, USA).

## Results

A total of 534 subjects registered their interest in joining the survey. There were 527 (98.7%) appropriate recipients, excluding 7 email addresses recognized as spam or with invalid domains.

### Demographics

In total, 388/527 (73.6%) responded to the survey. Respondents were mostly women (61%), between 30 and 39 years of age (52%), physicians (74%), with permanent contract (68%), practicing in sectors with standard risk of infection (79%), at academic (29%) or non-academic hub (38%) hospitals, from high-prevalence regions (52%) (Table 1).

**Table 1:** Respondents’ demographics (N = 388)

	N (%)
Gender	
Males	153 (39.4)
Females	235 (60.6)
Age group (years)	
< 30	43 (11.1)
30–39	200 (51.6)
40–49	81 (20.9)
50–59	51 (13.1)
≥ 60	13 (3.3)
Regional distribution (No. COVID-19 cases/100,000 people)^a^	
≥ 200 (N = 8 regions)	200 (51.5)
< 200 (N = 12 regions)	188 (48.5)
Type of healthcare worker	
Physician	287 (74.0)
Other^b^	101 (26.0)
Type of employment contract Permanent	262 (67.8)
Fixed-term	29 (7.5)
Temporary	35 (9.0)
Self-employed	61 (15.7)
Specialty sectors and risk of infection	
High (N = 6)^d^	82 (21.1)
Standard (N = 40)	306 (78.9)
Type of workplace	
Academic hospital	111 (28.6)
Non-academic hub hospital	148 (38.1)
Non-academic spoke hospital	46 (11.9)
General practice clinic	24 (6.2)
Private clinic	16 (4.1)
Other^c^	43 (11.1)

^a^Population prevalence on April 4th 2020 (survey closing date)

^b^Includes nurses, social health workers, pharmacists, and hospital administrative staff

^c^Includes home care support clinics, community pharmacies and health districts

^d^Includes the followings: intensive care unit, pneumology, infectious diseases, emergency medicine, microbiology, radiology

### Respondents’ Health Status and COVID-19 Screening

Only 16% of respondents admitted health problems in their medical history requiring chronic drug therapy and 33% received influenza vaccine in the past 6 months (Table 2). The latter observation substantially differ by age groups (P = 0.589).

**Table 2:** Respondents’ health status and characteristics of working environment

	N (%)
Received influenza vaccine in the season 2019–20	128 (33.0)
Health problems requiring chronic drug therapy	63 (16.2)
Presence of at least one typical symptom (fever, dry cough, myalgia) in the past 14 days	95 (24.5)
COVID-19 positive cases that respondents have come in close contact with	
Patients within the working centre	370 (95.4)
Patients within the working unit	219 (56.4)
Colleagues within the working unit	186 (47.9) 101 (26.0)
Family members or friends	
Involvement in the extraordinary management of COVID-19 patients with tasks beyond respondent’s own specialty	87 (22.4)
Indications for COVID-19 screening at the workplace	
No screening is planned for healthcare workers	122 (31.4)
Screening occurs if symptomatic or close contact with COVID-19 cases	217 (55.9)
All healthcare workers are screened	49 (12.6)
Respondents tested for COVID-19	98 (25.3)
More than once	39 (39.8)
Due to symptoms	6 (15.4)
Due to local screening policy in absence of symptoms	28 (71.8)
Due to a new close contact at risk	5 (12.8)
Symptomatic at the time of first testing	33 (33.7)
Testing positive for COVID-19	18 (4.6)
At first testing	13 (72.2)
At second testing	1 (5.6)
At third testing	4 (22.2)
Infection did likely occur while working	
Yes	16 (88.9)
No	1 (5.6)
Uncertain	1 (5.6)
Regular use of personal protective equipment	
No	6 (33.3)
Yes, everyone entering the workplace	9 (50.0)
Yes, but only the respondent	3 (16.7)
Use of medical therapy	11 (61.1)
Specific therapy for COVID-19	6 (54.5)
NSAIDs	2 (18.2)
Both	5 (45.4)
Required hospital admission	1 (5.6)
Required O_2_-therapy	1 (5.6)
Quarantined	42 (10.8)
Readily availability of personal protective equipment	298 (76.8)
Quantity and quality rating	
Adequate	64 (21.5)
Partially adequate	73 (24.5)
Inadequate	161 (54.0)
Number of intensive care unit beds before the outbreak	
< 5	20 (5.1)
5–10	70 (18.0)
11–15	98 (25.3)
> 15	123 (31.7)
I do not know	77 (19.9)
Number of intensive care unit beds during COVID-19 emergency at the workplace	
Increased	317 (81.7)
With increase in dedicated staffing	133 (42.0)
Without increase in dedicated staffing	80 (25.2)
Remained unaltered	31 (8.0)
Activation of local protocols for management of COVID-19 patients	336 (86.6)
Personally contributed to its development	66 (19.6)
Comply with its requirements	204 (60.7)
Deaths related to COVID-19 occurred at the workplace	247 (63.7)
Management was correct, with cause of death most likely resulting from respiratory failure	
Yes	114 (46.1)
No	33 (13.4)
I do not know	100 (40.5)
Number of deaths likely resulting from suboptimal bed capacity	
More than 50%	11 (4.4)
Less than 50% but still significant	34 (13.8)
Very few	47 (19.0)
None	155 (62.8)
Number of deaths likely resulting from non-compliance to protocols	
More than 50%	21 (8.9)
Less than 50% but still significant	26 (10.5)
Very few	96 (38.8)
None	104 (42.1)
10-point Likert scale rating (1 = extremely poor; 10 = excellent) of the local management of COVID-19 emergency, mean (standard deviation)	5.7 (1.8)
Perceive psychological safety	
Over the last few weeks	77 (19.8)
Currently	97 (25.0)
Believe to have been the source of infection	
For patients	29 (7)
For work colleagues	35 (9)
For family members	33 (9)
At least one work colleague died from COVID-19	27 (7.0)
At least one family member or friend died from COVID-19	46 (11.9)
Believe that psychological support for healthcare workers is useful during COVID-19 emergency	247 (63.7)
Psychological support available at the workplace	187 (48.2)
Currently receiving psychological support	13 (3.3)
Workload over last few weeks	
Decreased	162 (41.8)
Unaltered	56 (14.4)
Slightly increased	58 (15.0)
Moderately increased	61 (15.7)
Extremely increased	32 (8.2)
Increased to the extreme of own strengths	19 (4.9)

One fourth (25%) experienced typical symptoms of COVID-19 infection during the last 14 days prior to survey completion. The majority of respondents (95%) reported to have been in close contact with confirmed COVID-19 patients within their working centre. A smaller proportion admitted close contact with positive patients (57%) or work colleagues (48%), and 26% with positive family members or friends.

Only 13% of respondents stated that screening was routinely planned for the whole staff. Among these, statistically significant higher proportion of HCW came from high- rather than low-prevalence regions (84% vs. 16%, respectively; P < 0.001). Moreover, almost one third (31%) of respondents declared that no screening plan for operators was in place at their workplace.

In total, 98 (25%) respondents underwent recent testing for COVID-19 by nasopharyngeal swab, with one third admitting typical symptoms at the time of screening. Among the tested population, 18 (18%) HCW resulted positive for COVID-19, mostly at first testing (72%), with contagion likely occurring while working (89%). Eleven (61%) required medical therapy and 1 hospital admission. One third of COVID-19 positive HCW declared to be asymptomatic. Among the 12 symptomatic, those who did not receive influenza vaccine in the past 6 months had experienced a longer length of symptoms (beyond 10 days) compared to those who did (P = 0.015).

Prevalence of tested and/or COVID-19 positive HCW was similar between high- and low-prevalence regions, with homogeneous distribution within age groups and professional sectors. Those reporting typical symptoms during the last two weeks were more likely – but not statistically significant—to come from high-prevalence regions (OR, 1.48 [CI 95% 0.93–2.37]; P = 00,098). Despite being more likely tested for COVID-19 (adjusted OR 3.61, CI 95% 2.15–6.06; P < 0.001) compared to asymptomatic, less than a half (45%) of symptomatic HCW was actually screened for COVID-19. Most HCW who were tested for COVID-19 or resulted positive denied to be working in dedicated COVID units.

Quarantine was more frequently activated for symptomatic HCW (OR, 6.61 [CI 95% 3.09–14.16]; P < 0.001) or those tested for COVID-19 (OR, 8.29 [CI 95% 3.81–18.01]; P < 0.001), regardless of regional provenience.

### Personal Protective Equipment (PPE)

Although most respondents (77%) confirmed that PPE were readily available at the workplace, only 22% considered PPE adequate for quality and quantity. PPEs were more readily available in high-risk specialty sectors (OR, 1.96 [CI 95% 0.98–3.94]; P = 0.058) but less likely for HCW with recent onset of symptoms (OR, 0.48 [CI 95% 0.28–0.83]; P = 0.009). Furthermore, respondents involved in the extraordinary management of COVID-19 patients stated that PPE were more readily available (87.4%) compared to those working in standard care units (P = 0.012).

Six (33%) out of the 18 positive HCW denied regular use of PPE at the time of possible contagion. In 2 (11%) cases, PPE were not readily available at the workplace.

### Management Strategies

Overall, 57% of respondents indicated that intensive care bed capacity at their (or referring) hospital prior to the outbreak exceeded 10 units. However, bed capacity was statistically significant lower in low-prevalence regions (P < 0.001) and in spoke hospitals (P < 0.001), compared to regions with high-prevalence and hub and academic centres, respectively. A total of 317 (81.7%) respondents reported an increase in bed capacity related to COVID-19 emergency, which was nevertheless accompanied by an increase in dedicated staffing in only 42% of cases. Implementation of intensive care units was significantly reduced in low- compared to high-prevalence regions (P < 0.001) and in spoke compared to academic and hub centres (P < 0.001).

Over one fifth (22%) of HCW were being involved in the extraordinary management of COVID-19 patients with tasks beyond their own specialty. Distribution of symptomatic, tested, and COVID-19 positive respondents did not substantially differ between this group of HCW and those continuing standard practices.

Most respondents (87%) reported activation of local protocols for management of COVID-19 patients at their workplace, with one fifth personally contributing to its development.

Slightly less than two third of HCW (N = 247 [64%]) stated that deaths related to COVID-19 occurred at their workplace. In high-prevalence regions, a statistically significant higher number of HCW (71%) confirmed COVID-19 death occurrence compared to those who did not (29%; P = 0.002), as opposed to low-prevalence regions, with respondents more homogeneously distributed. A minority of HCW (13%) did not consider respiratory failure as the main cause of death, which was rather deemed related to suboptimal bed capacity or non-compliance to protocols.

**Fig. 2:**
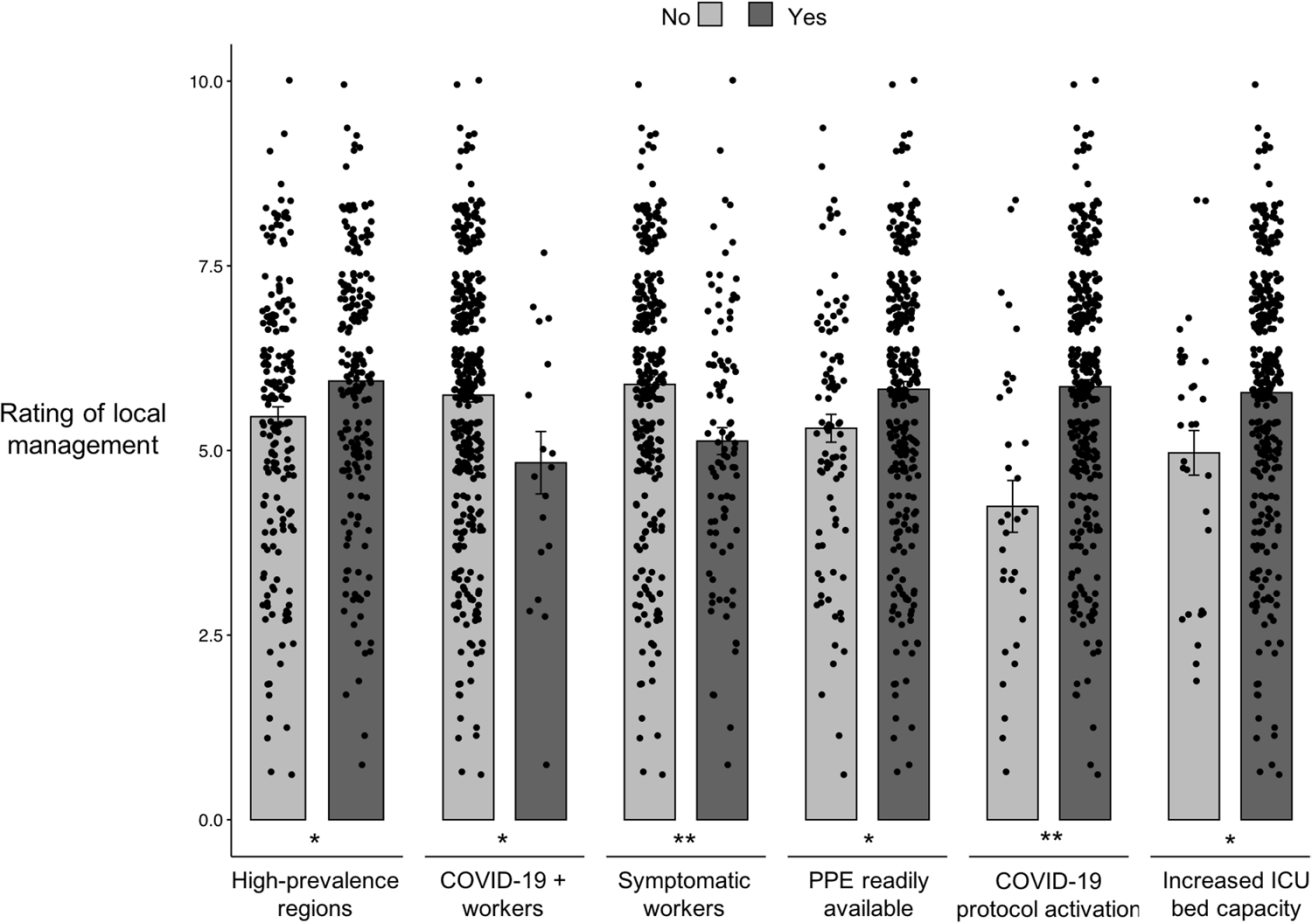
Subgroup comparisons in mean scores on a 10-point Likert scale rating (1 = extremely poor; 10 = excellent) of the local management of COVID-19 emergency. *P < 0.05; **P < 0.001. HCW: healthcare workers; PPE: personal protective equipment; ICU: intensive care unit

The average rate for local management of COVID-19 emergency on a 10-point Likert scale was 5.7 (standard deviation, 1.8). Significantly higher scores were reported by HCW from high-prevalence regions (P = 0.008) or confirming PPE readily availability (P = 0.024), whilst those complaining of recent symptoms (P < 0.001) or testing positive for COVID-19 (P = 0.010), as well as those from centres where protocols had not been developed (P < 0.001) nor bed capacity increased (P = 0.033), reported lower scores (Fig. 2). Nevertheless, similar scores were recorded by HCW reporting or not COVID-19 death occurrence (P = 0.237).

### Psychological Support and Workload

Only 20% and 25% of HCW declared to feel psychologically safe over the last few weeks and at the time of survey completion, respectively. Nearly 10% of respondents believed to have been the source of infection for work colleagues or family members. Furthermore, 7% and 12% of respondents reported the loss of a work colleague and a family member or friend for COVID-19, respectively. Despite being considered useful by most (64%), less than 50% of HCW had access to psychological support if needed (48%). A higher proportion of HCW felt safe in centres offering psychological support (62% vs. 38% when unavailable; P = 0.002) and PPE readily availability (31% vs. 7%; P < 0.001). Only 3% of HCW was receiving support at the time of survey completion.

As compared to their counterparts, females (OR, 1.78 [CI 95% 1.14–2.78]; P = 0.012) and respondents working in high-risk sectors (OR, 2.02 [CI 95% 1.12–3.65]; P = 0.020) were more likely to rate psychological support as useful, as opposed to the oldest HCW compared to the youngest (OR, 0.22 [CI 95% 0.05–0.95]; P = 0.045) (Table 3).

**Table 3:** HCW believing that psychological support is useful: associations with the covariate set according to hierarchical logistic model

		OR	SE	*P*	95% CI	
					Lower	Upper
Age (years)	< 30	Ref				
	30–39	1.12	0.42	0.754	0.54	2.32
	40–49	1.08	0.44	0.852	0.48	2.42
	50–59	0.73	0.32	0.484	0.31	1.74
	≥ 60	0.22	0.16	0.042	0.05	0.95
Gender	Males	Ref				
	Females	1.78	0.41	0.012	1.14	2.78
Specialty sector	Standard risk	Ref				
	High-risk	2.02	0.61	0.020	1.12	3.65
Type of HCW	Other	Ref				
	Physician	0.85	0.23	0.562	0.50	1.45

*Ref* Reference category, *OR* odds ratio, *SE* standard error, *CI* confidence interval, *HCW* healthcare worker

Workload was reported as decreased by 42%, unaltered by 14%, and increased by 44% of HCW. The latter rating was less frequently stated by physicians (OR, − 0.51 [CI 95% − 0.87 to − 0.14]; P = 0.007) as opposed to female respondents (OR, 0.38 [CI 95% 0.06–0.69]; P = 0.018) and HCW practicing in high-risk sectors (OR, 0.54 [CI 95% 0.16–0.92]; P = 0.005) (Table 4).

**Table 4:** Increased workload: associations with the covariates set according to the mixed model

		Mean increase	SE	*P*	95% CI	
					Lower	Upper
Age (years)	< 30	Ref				
	30–39	– 0.37	0.25	0.149	– 0.87	0.13
	40–49	– 0.33	0.28	0.252	– 0.88	0.23
	50–59	– 0.02	0.31	0.954	– 0.63	0.59
	≥ 60	0.54	0.48	0.259	– 0.40	1.48
Gender	Males	Ref				
	Females	0.38	0.16	0.018	0.06	0.69
Specialty sector	Standard risk	Ref				
	High-risk	0.54	0.19	0.005	0.16	0.92
Type of HCW	Other	Ref				
	Physician	– 0.51	0.19	0.007	– 0.87	–0.14

*Ref* Reference category, *SE* standard error, *CI* confidence interval, *HCW* healthcare worker

## Discussion

### Key Results

This is the first study to examine the impact of COVID-19 emergency on HCW in Italy. The survey demonstrated profound variations across high- and low-prevalence regions, specialty sectors and professional figures. Most respondents were physicians and the response rate (74%) was far higher than previously reported from surveys in this population (i.e. usually not exceeding 20%) [10].

### Limitations

Despite the survey being launched nationally including using social media, it did not obtain significant response from HCW in a number of regions. However, using the arbitrary cut-off of 200 cases per 10^5^ people, respondents resulted quite homogeneously distributed between low- and high-prevalence regions. A number of professional figures were numerically poorly represented (e.g. social health workers) thus hampering generalizability of the results. Despite the small number in the surveyed population, prevalence of COVID-19 positive cases well mirrored current national estimates.

### Interpretation

PPE were less likely deemed readily available by HCW reporting recent onset of symptoms (OR, 0.48 [CI 95% 0.28–0.83]; P = 0.009), indicating that they may have been a vehicle for transmission to patients, work colleagues or family members. The Italian decree March 9th 2020 establishes that quarantine does not apply to HCW, who should stop working—in line with our results—only if they became symptomatic or COVID-19 positive [11]. These government indications may be valid upon confirmed readily availability of PPE for HCW.
